# Editorial: Digital health innovations in Africa: harnessing AI, telemedicine, and personalized medicine for improved healthcare

**DOI:** 10.3389/fphar.2026.1799832

**Published:** 2026-02-10

**Authors:** Rohan M. Benecke, Innocent G. Asiimwe, Ahmed A. Abulfathi, Samer Mouksassi, Bernd Rosenkranz

**Affiliations:** 1 Division of Clinical Pharmacology, Faculty of Medicine and Health Sciences, Stellenbosch University, Cape Town, South Africa; 2 Department of Pharmacology and Therapeutics, Wolfson Centre for Personalized Medicine, University of Liverpool, Liverpool, United Kingdom; 3 Certara USA, Inc., Radnor, PA, United States; 4 Department of Bioengineering and Therapeutic Sciences, University of California San Francisco, San Francisco, CA, United States; 5 Fundisa African Academy of Medicines Development, Cape Town, South Africa; 6 Institute for Clinical Pharmacology and Toxicology, Charité – Universitätsmedizin Berlin, Berlin, Germany

**Keywords:** Africa, artificail intelligence (AI), digital health, pharmacology, telemedicine

## Introduction

The healthcare landscape in Africa is undergoing a transformative shift driven by the integration of Artificial Intelligence (AI), telemedicine, and advanced computing. This Research Topic, an initiative linked to the Digital Health Africa 2024 Conference (Benecke et al.), brings together a Research Topic of studies that examine how these technologies address complex healthcare challenges exacerbated by underfunding and resource limitations. While the potential for innovation is immense, the contributions highlight a recurring tension: the gap between policy aspirations and the current reality of infrastructure.

## AI as a catalyst for efficiency in research and education

One of the most immediate impacts of AI is its ability to reduce the manual burden of labour-intensive tasks. Abogunrin et al. provide a pragmatic review quantifying the workload efficiencies of AI in evidence synthesis, especially for systematic literature reviews. Their findings indicate that AI can cut overall processing time by more than 50% and reduce abstract screening time by a factor of 5–6, thereby accelerating the pace at which evidence-based medicine informs policymaking.

## Beyond research, AI is reshaping professional training

Almaghaslah investigates the use of ChatGPT-4 in pharmacy education to combat the global phenomenon of grade inflation. This study demonstrates that AI-generated multiple-choice questions yield higher reliability (KR-20 = 0.83) than those generated by human (KR-20 = 0.78). By generating more moderate and difficult questions, AI helps ensure that student evaluations accurately reflect true mastery rather than superficial learning.

## Bridging the last mile in healthcare delivery

Implementation in resource-limited settings remains the final Frontier for digital health. Two studies in this Research Topic offer critical insights into the operationalization of these tools in Sub-Saharan Africa. Nkangu et al. describe the integration of the World Health Organization’s (WHO) Digital Adaptation Kit (DAK) for antenatal care into the BornFyne-Prenatal Management System in Cameroon. By utilizing “machine-readable” guidelines, the project ensures that digital health interventions are not only innovative but also standardized and interoperable with preexisting systems like District Health Information Software 2 (DHIS2).

However, technological readiness is often stymied by physical realities. Stenhouse et al. analysed the development of a mobile health (mHealth) system for symptomatic management of COVID-19 in rural Ethiopia. They identified infrastructure and digital access, specifically unreliable electricity and internet outages, as primary barriers to sustainable implementation. The authors also note clinicians’ concerns that remote monitoring could miss critical findings detectable only through physical examination.

## Optimizing supply chains and regulatory frameworks

The logistical hurdles of vaccine distribution in Africa, characterized by “last mile” visibility gaps and cold chain failures, present another opportunity for AI intervention. Musa et al. discuss the leverage of AI for predictive analytics to optimize vaccine stock levels and delivery routes. They highlight successful implementations of autonomous drone delivery services, such as Zipline, in Rwanda and Ghana, which have significantly reduced delivery times and improved vaccine availability even in remote locations.

As these technologies proliferate, the need for robust regulatory oversight becomes urgent. While global bodies like the Food and Drug Administration (FDA), European Medicines Agency (EMA), and the National Institute for Health and Care Excellence (NICE) are establishing guiding principles for AI, local implementation remains fragmented. In this Research Topic, Abogunrin et al. highlight how the NICE AI position statement provides a foundation for evidence generation, while Botes examines the specific regulatory challenges in South Africa, focusing on mental health applications. The study warns of inadequacies in the regulation regarding the commodification of sensitive neural data and the ethical risks posed by AI-driven tools that lack clinical validation. Without explicit guidelines for third-party data sharing and AI transparency, users remain vulnerable to privacy breaches and psychological harm.

## Discussion: the human-in-the-loop and future directions

A common thread across all submissions is the indispensable role of human oversight. Whether it is the meticulous review required to ensure the accuracy of AI-generated exam questions or the structured protocols needed for automated evidence synthesis, technology cannot yet replace the nuanced judgment of a human professional.

Furthermore, the digital gap remains a significant obstacle. For digital health to be equitable, interventions must confront low digital and health literacy levels prevalent in many rural communities. Successful implementation therefore requires a bottom-up approach that involves healthcare providers and patients from the planning stage onwards, ensuring interventions are culturally relevant and contextually appropriate.

## Conclusion

The articles in this Research Topic collectively demonstrate that Digital Health innovations in Africa offer a promising path toward universal health coverage and more resilient supply chains, bridging the gap between advanced technological capabilities and the continent’s physical and systemic realities. However, technology alone is not a panacea. Realizing their full potential requires strategic frameworks that balance innovation with rigorous data protection and substantial government investment in digital infrastructure. Only by grounding Africa’s transformative healthcare shift on reliability, ethics, and sustainability can we secure lasting impact.

